# Effects of reduced nitrogen inputs on crop yield and nitrogen use efficiency in a long-term maize-soybean relay strip intercropping system

**DOI:** 10.1371/journal.pone.0184503

**Published:** 2017-09-14

**Authors:** Ping Chen, Qing Du, Xiaoming Liu, Li Zhou, Sajad Hussain, Lu Lei, Chun Song, Xiaochun Wang, Weiguo Liu, Feng Yang, Kai Shu, Jiang Liu, Junbo Du, Wenyu Yang, Taiwen Yong

**Affiliations:** 1 College of Agronomy, Sichuan Agricultural University, Chengdu, P.R China; 2 Sichuan Engineering Research Center for Crop Strip Intercropping System, Key Laboratory of Crop Ecophysiology and Farming System in Southwest China (Ministry of Agriculture), Sichuan Agricultural University, Chengdu, P. R. China; 3 Renshou Weather Bureau, Meishan, P.R. China; Agroecological Institute, CHINA

## Abstract

The blind pursuit of high yields via increased fertilizer inputs increases the environmental costs. Relay intercropping has advantages for yield, but a strategy for N management is urgently required to decrease N inputs without yield loss in maize-soybean relay intercropping systems (IMS). Experiments were conducted with three levels of N and three planting patterns, and dry matter accumulation, nitrogen uptake, nitrogen use efficiency (NUE), competition ratio (CR), system productivity index (SPI), land equivalent ratio (LER), and crop root distribution were investigated. Our results showed that the CR of soybean was greater than 1, and that the change in root distribution in space and time resulted in an interspecific facilitation in IMS. The maximum yield of maize under monoculture maize (MM) occurred with conventional nitrogen (CN), whereas under IMS, the maximum yield occurred with reduced nitrogen (RN). The yield of monoculture soybean (MS) and of soybean in IMS both reached a maximum under RN. The LER of IMS varied from 1.85 to 2.36, and the SPI peaked under RN. Additionally, the NUE of IMS increased by 103.7% under RN compared with that under CN. In conclusion, the separation of the root ecological niche contributed to a positive interspecific facilitation, which increased the land productivity. Thus, maize-soybean relay intercropping with reduced N input provides a very useful approach to increase land productivity and avert environmental pollution.

## Introduction

With the continuous increase in the global population, food security problems are increasing, particularly in China and India with 37% of the world population [1]. In China, food production has greatly improved with the increased application of chemical N fertilizer. However, sustainable agricultural is attracting increased interest due to the depletion of fossil fuels and problems with food security. Industrially produced N fertilizer increases the environmental costs of agricultural production and environmental pollution [2]. Additionally, the abuse of N fertilizer further increases the environmental cost and decreases N use efficiency [3]. The inputs of chemical N fertilizer can be reduced by breeding N efficient cultivars, optimizing N nutrient management and choosing a suitable cropping system [4–6].

Intercropping and relay intercropping are used globally as sustainable practices, e.g., in China, India, Southeast Asia, Latin America, and Africa. These practices use land efficiently, are high-yield, and provide efficient control of weeds, diseases and pests [7, 8]. Environmental resources, such as heat and light, can limit cropping systems, but with sufficient contributions of these resources, annual crop harvests increase. Heat and light resources are used efficiently with the practices of intercropping and relay intercropping to increase land output. Maize-soybean intercropping is used in areas with two crops a year (or three crops), e.g., the Huang-Huai-Hai region and in northwest China [9], whereas in areas with one crop a year (or three crops in two years), e.g., in southwest China [10], maize-soybean relay intercropping is the practice. In relay intercropping systems, the behaviors of component crops differ from those in sole cropping, and the grain yield and NUE are also affected. In a previous study, relay intercropping with legumes significantly increased the N uptake of the subsequent crop, leading to a 30% increase in grain yield [11]. Compared with the corresponding monocultures, overall nitrogen resources are used 30–40% more efficiently in legume-cereal intercropping [12]. Maize-soybean relay intercropping increases farm land productivity (i.e., the land equivalent ratio of maize-soybean relay intercropping systems ranges from 1.61 to 1.59), in contrast to the monocultures [13]. According to Yamane et al., the land equivalent ratio of relay intercropping is higher than that of double cropping in a legume cropping system [14].

However, most studies focus on relay intercropping systems in which the legumes play a secondary role, i.e., the legumes are used as a cover crop, and the legume yield is not considered. Previous studies show that facilitation and competition coexist in intercropping systems, particularly in legume/non-legume intercropping systems [2]. Xia et al. reported that legumes facilitate the root system and grain yield of maize considerably [13], whereas Fan et al. found that the grain yield of fava bean decreased in a wheat/fava bean intercropping system in contrast with a monoculture [2]. Generally, the architecture of a plant influences the relative competitive ability. Soybean seedlings often grow under the shade of the maize canopy and then are transferred to full sunlight after the harvest of maize [15]. Moreover, the distribution of the root system plays a key role in the acquisition of belowground nutrients. The roots of cereals occupy soils both near the surface and in deeper layers, whereas the roots of legumes are distributed in the upper soil layers [16]. The competitive ability of cereals for soil N is stronger than that of legumes in an intercropping system [17]. Furthermore, the separation of the root ecological niche between component crops is well known to affect the total grain yield of intercropping systems. In a maize/fava bean system compared with that of a wheat/fava bean system, the total grain yield was significantly higher [2]. Nitrogen recovery efficiency and uptake efficiency can increase significantly through the interaction of roots between crops. Furthermore, the bi-directional N transfer and positive N competition between crops are advantageous to improve to NUE in the wheat/maize/soybean relay intercropping system [18].

Early studies demonstrated that the land equivalent ratio (LER) of maize-soybean relay intercropping is greater than 1 [19], namely, maize-soybean relay intercropping can increase land productivity. However, previous studies focused on resource utilization in maize-soybean relay intercropping, and the distribution of roots belowground remains unclear [12, 19, 20]. Little information is available on the effect of reduced N on the yield advantage and increase in NUE in relay intercropping systems. Additionally, no evidence is available on the effect of reduced N inputs on facilitation in maize-soybean relay strip intercropping. Therefore, the aims of this study were (i) to evaluate the effect of reduced N input on crop grain yield and NUE in the maize-soybean relay strip intercropping system, and (ii) to assess the effect of reduced N input on crop roots distribution in the maize-soybean relay strip intercropping system, and (iii) to analyze the effects of crop roots distribution on crop growth in the maize-soybean relay strip intercropping system.

## Materials and methods

### Ethics statement

No specific permits were required for the described field studies. All experiments were performed according to institutional guidelines of Sichuan Agricultural University, China.

### Description of study sites

Experiments were conducted from 2012 to 2014 at two sites in Sichuan Province, China, as detailed below.

#### Experiment 1

**Fig 1:**
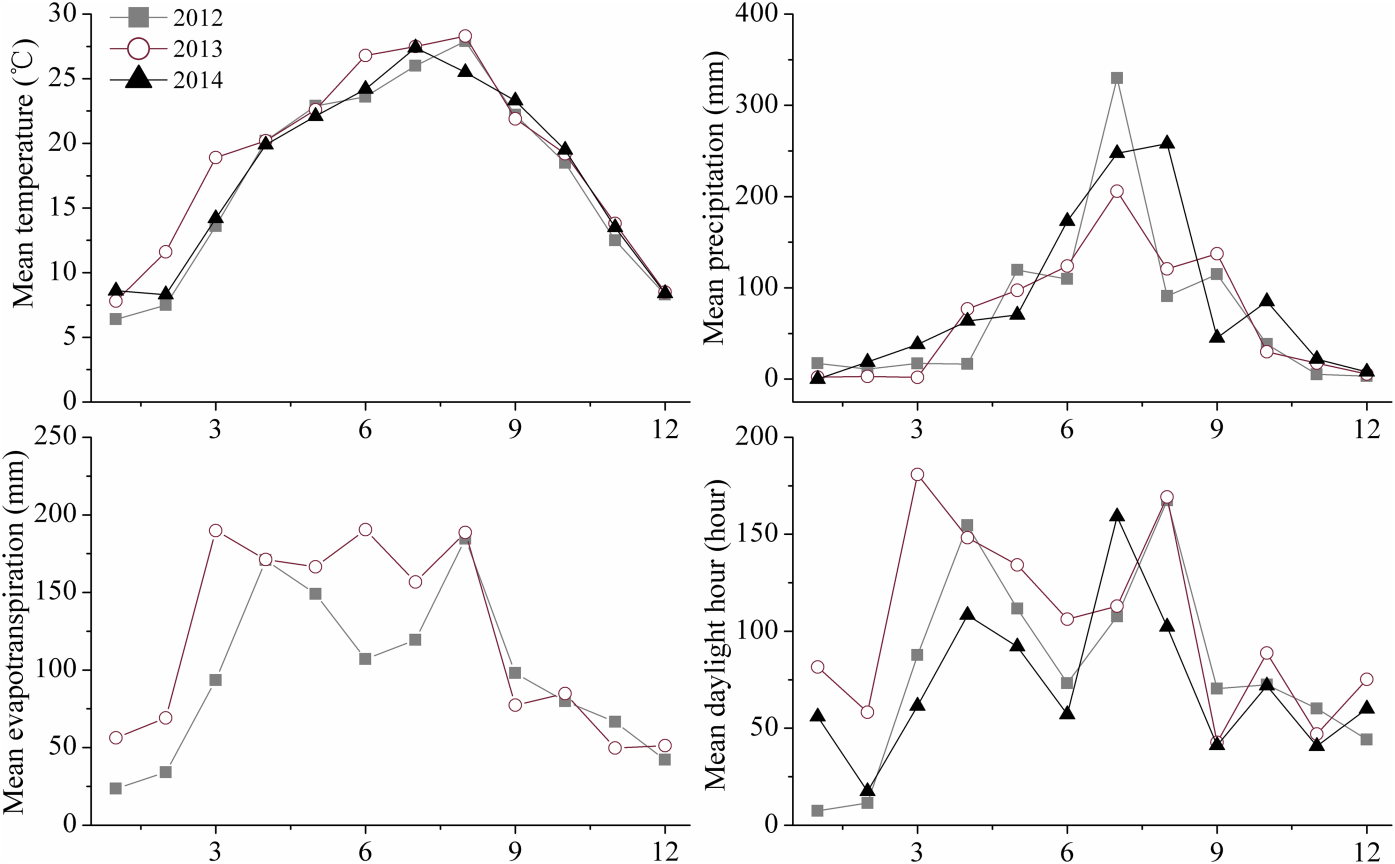
The temperature, daylight hour, precipitation and evapotranspiration during the cropping seasons from 2012 to 2014.

The experiment was conducted in Renshou County (29°60' N, 104°00' E), Sichuan Province, China. The field climate was subtropical monsoon humid, with average annual temperature of 17.4°C, rainfall of 1009.4 mm, and sunshine of 1196.6 hours. Fig 1 shows the temperature, daylight hours, precipitation and evapotranspiration for the cropping seasons. Total N, total P, total K, alkali hydrolysable N, Olsen-P and exchangeable K in the top 20 cm of soil of the experimental site were 0.90 g kg^-1^, 0.50 g kg^-1^, 14.28 g kg^-1^, 77.35 mg kg^-1^, 22.83 mg kg^-1^, and 196.63 mg kg^-1^, respectively.

The long-term field experiment consisted of three planting patterns, i.e., monoculture maize (MM), monoculture soybean (MS), and maize-soybean relay strip intercropping (IMS), and three application rates of total nitrogen, i.e., no nitrogen (NN), reduced nitrogen (RN) of 180 kg N ha^-1^, and conventional nitrogen (CN) of 240 kg N ha^-1^. The compact maize (*Zea mays* L. cv. Denghai-605) and shade-tolerant soybean (*Glycine max* L. cv. Nandou-12) were used as experimental crops. Maize was sown on April 1, 2012, April 3, 2013, and April 5, 2014; and harvested on July 29, 2012, August 1, 2013, and August 2, 2014, respectively. Soybean was sown on June 10, 2012, June 11, 2013, and June 15, 2014, with simultaneous application of maize topdressing and soybean base fertilizer; and harvested on October 31, 2012, October 29, 2013, and October 26, 2014, respectively.

**Fig 2:**
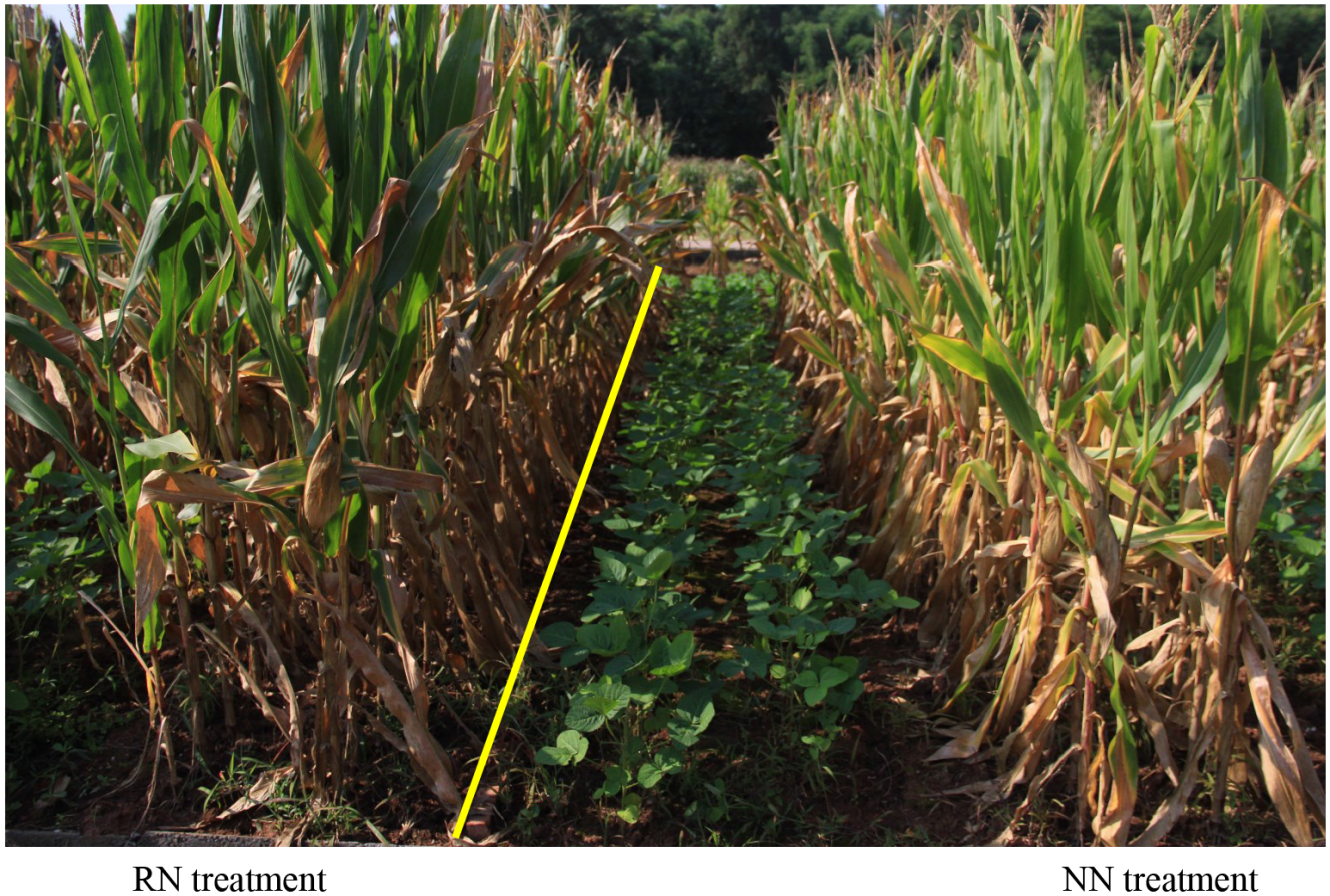
Maize-soybean relay intercropping system in August. Left side is RN treatment plot, right side is NN treatment, and the yellow line is the plot boundary.

Monoculture was planted with row spacing and plant density for maize (MM) of 1.0 m and 58,500 per hectare (ha.) and for soybean (MS) of 0.5 m and 117,000 per ha. The plant spacing was 17 cm for all treatments, with a post-emergence density of 1 maize and 1 soybean plant per hole for the corresponding monocultures. The post-emergence density was 1 maize and 2 soybean plants per hole for maize-soybean relay strip intercropping. The plant density per unit area was equal for intercropping and the corresponding monocultures. All plots were planted with three strips that were 6 m in length and 2 m in width. The total number of plots was 27. In the maize-soybean relay strip intercropping system (IMS), a wide-narrow row planting (160 cm for wide rows and 40 cm for narrow rows) was adopted, resulting in a total ratio of maize to soybean rows of 2:2. Maize plants (IM) were in the narrow rows with row spacing of 40 cm, and soybean plants (IS) were in the wide rows with row spacing of 40 cm. Additionally, the distance between maize and soybean rows was 60 cm (Fig 2).

Potassium chloride, superphosphate, and urea were used as P, K and N fertilizers, respectively. The N fertilizer for maize was divided into two applications, 72 kg N ha^-1^ for base fertilizer and the rest for topdressing. The P and K fertilizers were used as base fertilizers at 105 kg P_2_O_5_ ha^-1^ and 112.5 kg K_2_O ha^-1^ for maize and 63 kg P_2_O_5_ ha^-1^ and 52.5 kg K_2_O ha^-1^ for soybean, respectively. All fertilizers were base placement, except for the RN treatments in the maize-soybean relay strip intercropping system. In RN and CN, the N fertilizer for maize was divided into two applications, 72 kg N ha^-1^ for base fertilizer and the rest for topdressing. Under RN treatment, the base N fertilizer for IM was base placement. The N topdressing for IM was integrated with the soybean base fertilizer and strip placement, with a distance of 25 cm from maize rows to soybean rows in IMS (the optimizing of fertilization methods, unpublished data).

#### Experiment 2

The intercropping advantages in grain yield and N utilization identified in Experiment 1 suggested that belowground facilitation might be responsible for the aboveground growth and efficient use of nutrients. Experiment 2 was conducted with the same three planting patterns and rates of total nitrogen application as in experiment 1. The planting density and fertilization methods and amounts were same as in Experiment 1; however, the row spacing was different, which was 60 cm for all treatments. This experiment was conducted within a rhizo-box, which had a length, width, and height of 1 m, 0.38 m and 1 m, respectively. Each rhizo-box was planted with 2 rows and 2 holes per row, with row spacing and plant spacing of 0.6 m and 0.17 m, respectively. IMS was planted with 2 maize and 4 soybeans; MM was planted with 4 maize plants; and MS was planted with 8 soybeans.

### Determination of crop dry matter and N uptake

In field experiment 1, samples were collected in the middle row of each plot, and the crop grain yield and straw and root dry matter at physiological maturity were determined. Crop roots were collected from soil blocks, with the length and depth 34 cm (dug in the middle of two plants in a row) and 40 cm, respectively, and the width (dug in the middle of two crop rows) 100 cm for MM, 50 cm for IM, and 50 cm for MS and IS. After manual identification, root samples were hand-washed and oven-dried at 80°C for 72 h before weighing. Crop straw was oven-dried at 80°C for 72 h before weighing. Samples were weighed, and the N content of straw and grain was determined using the Kjeldahl method [21].

Data on root distribution in soil was obtained by washing off the soil on site [22], which was a time-consuming and labor-intensive operation to conduct in the field. In a previous study, the vertical distribution was determined using an auger to collect soil cores (5.5 cm diameter) at 10 cm intervals to a maximum depth of 100 cm [13]. We conducted the rhizo-box experiment to study crop root distribution, both during the coexistence period (at the V3 stage of soybean) and at the R2 stage of soybean growth [23]. Both the horizontal and vertical distributions of root systems were investigated (S1 Fig). Crop roots were collected from soil blocks, and after manual identification, root samples were hand-washed and oven-dried at 80°C for 72 h before weighing. Soil block length, width, and height were 10 cm, 0.38 m and 20 cm, respectively. Soil blocks were collected at 10 cm intervals in the horizontal direction and were centered on the crop stem base and sampled from maize row to soybean row in the coexistence period (or from soybean row to maize row at the R2 stage after maize harvest). In the vertical direction, the blocks were divided into depth increments of 20 cm (0–20, 20–40, 40–60, 60–80, and 80–100 cm for maize and 0–20 and 20–40 cm for soybean). Crop root contours were determined using surfer v. 8.0 (Golden Software Inc., Golden, CO, USA), and data-gridding was performed using a natural neighbor method [13].

Plant N uptake (NA, kg ha^-1^) was calculated as follows:

$$\text{NA }\left(\text{kg}\cdot {\text{ha}}^{-1}\right)\;=\text{ crop dry matter }\left({\text{kg ha}}^{-1}\right)\;\times \text{ crop N concentration }\left({\text{g g}}^{-1}\right)$$ (1)

The N use efficiency (NUE) was calculated as follows [24]:

$$\text{ NUE }\left(\text{ \%}\right)=\frac{U_{\text{MN}}+U_{\text{SN}}-U_{\text{M}0}-U_{\text{S}0}}{A_{\text{MN}}+A_{\text{SN}}}*100\;\%$$ (2)

where *U*_MN_ (or *U*_SN_) (kg N ha^-1^) is total N accumulation by maize (or soybean) with N application, *U*_M0_ (or *U*_S0_) is total N accumulation by maize (or soybean) without N application, and A_MN_ (or A_SN_) is the amount of N supplied during the growing season. The NUE of maize (or soybean) was calculated from formula (2) in which *U*_SN_ (or *U*_MN_), *U*_S0_ (or *U*_M0_) and A_SN_ (or A_MN_) were considered zero.

### Competition ratio and system productivity index

Competition ratio and system productivity index are used to assess interspecific competition and intercropping advantages, respectively. As an indicator, competition ratio is used to measure the degree of competition between crops in an intercropping system [25] and is calculated with the following formula:

$$CR_{\text{SM}}=\frac{Y_{\text{IS}}/\left(Y_{\text{MS}}\times A_{\text{S}}\right)}{Y_{\text{IM}}/\left(Y_{\text{MM}}\times A_{\text{M}}\right)}$$ (3)

where *CR*_*SM*_ is the competitive ratio of maize relative to soybean, *Y*_IS_ and *Y*_MS_ are the yield or nitrogen acquisition per unit area of maize under intercropping and monoculture, respectively, *Y*_IM_ and *Y*_MM_ are the yield or nitrogen acquisition per unit area of soybean under intercropping and monoculture, respectively, and *A*_S_ and *A*_M_ are the ratios of the area occupied by maize and soybean under the intercropping system relative to that of the corresponding monoculture, respectively. In this study, *A*_S_ and *A*_M_ were the same. A competition ratio greater than 1 indicated the competitive ability of soybean was greater than that of maize in the maize-soybean relay intercropping system. A ratio less than 1 indicated the competitive ability of soybean was less than that of maize in the maize-soybean relay intercropping system. System productivity index, SPI, is another indicator used to assess intercropping that standardizes the yield of the secondary crop in terms of the primary crop [26] and is calculated as follows:

$$\text{SPI}=\left(\frac{S_{\text{S}}}{S_{\text{M}}}\right)\times Y_{\text{M}}+Y_{\text{S}}$$ (4)

where *S*_S_ and *S*_M_ are the average yields of soybean and maize under monoculture, respectively, and *Y*_S_ and *Y*_M_ are the average yields of soybean and maize under intercropping, respectively.

### Statistical analyses

Data were analyzed with analysis of variance (ANOVA) using the SPSS v.22 [27], and the average values were compared using least significant differences (LSD) at the 5% level. Surfer v.8 was used to draw the figures of root distribution [13], and Origin Pro 8 was used to draw the figure.

## Results

### Competition ratio, land equivalent ratio, and system productivity index

**Fig 3:**
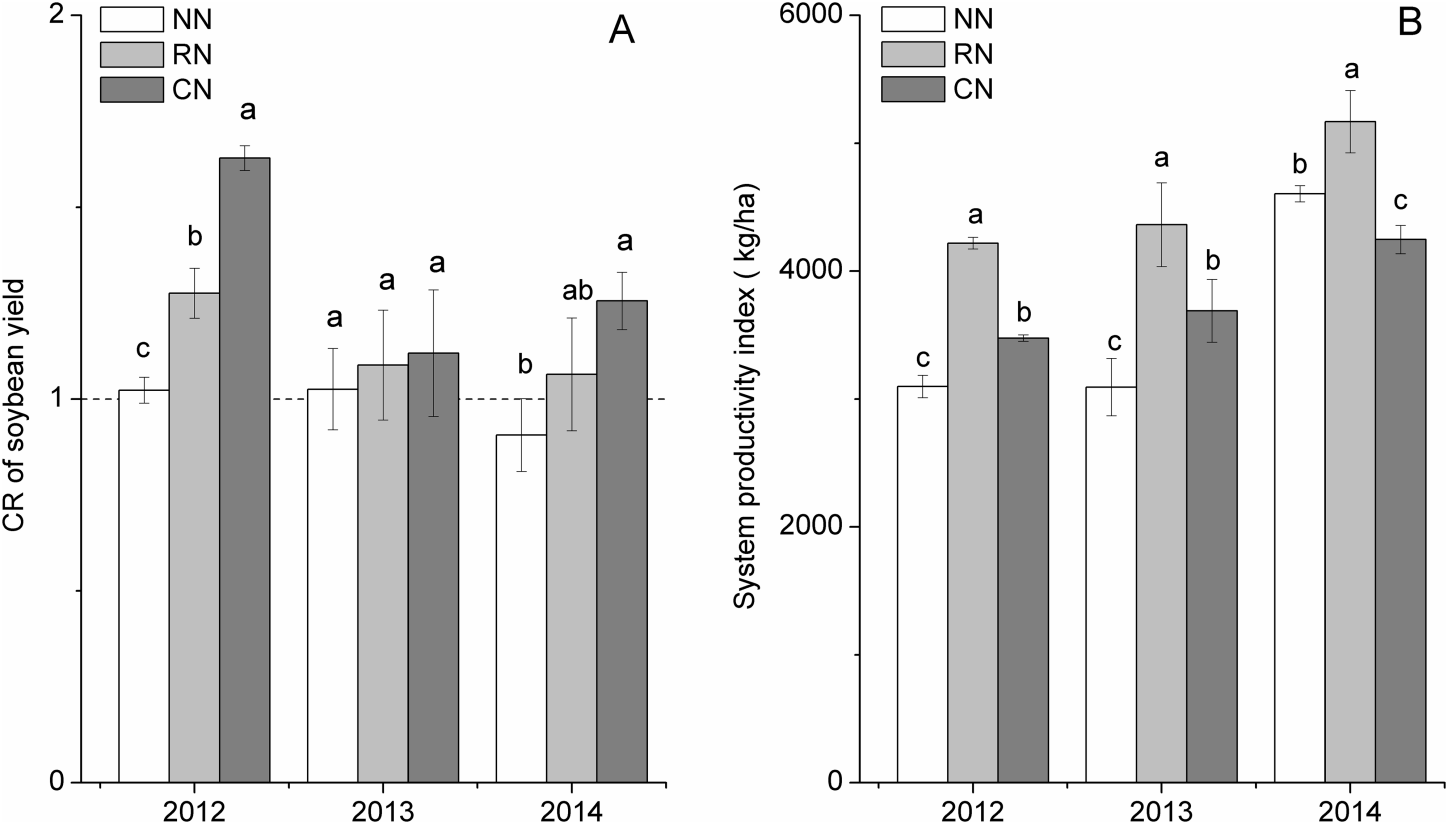
Effect of N application rates on competition ratio and system productivity index of the maize-soybean intercropping systems from 2012 to 2014. CR_SM_: yield competition ratio, SPI: system productivity index; Different lower case letters in the same column indicate significant differences (LSD, *P* < 0.05).

The level of N application affected the competition ratio of soybean relative to maize (CR_SM_) in the maize-soybean relay intercropping system (Fig 3). The CR_SM_ increased with the increase in N input, and the ratio in all treatments was greater than 1 in 2012, suggesting interspecific facilitation. Although the CR_SM_ decreased in 2013, the CR_SM_ of all treatments remained greater than 1. However, in 2014, the CR_SM_ of the NN treatment was less than 1, whereas that of RN and CN treatments was greater than 1. The trends for CR_SM_ indicated a positive interspecific interaction between component crops in the maize-soybean relay intercropping system, and N application was advantageous and improved the CR_SM_. With the increase in years of planting, the interspecific facilitation converted to interspecific competition in the NN treatment, although interspecific facilitation was retained under RN and CN, which suggested that N input was required to achieve interspecific facilitation in long-term maize-soybean relay intercropping.

The LER of all treatments was greater than 1, and the LER increased in RN and CN compared with that NN in the three years (Table 1). The LER of RN was lower than that of CN in 2012 but was higher than that of CN in 2013 and 2014. Based on crop yield, the SPI is a simple parameter that directly reflects the intercropping system productivity advantage, and the index increased continually from 2012 to 2014 (Fig 3). However, with increased N application rates, the SPI increased first and then decreased, and the SPI of RN was higher than that of CN by 21.35%, 18.28% and 21.71% in 2012, 2013 and 2014, respectively.

**Table 1:** Effects of N application rates on the land equivalent ratio (LER) of maize-soybean relay intercropping.

N levels	2012	2013	2014
NN	2.00±0.03c	1.85±0.14b	1.97±0.09a
RN	2.24±0.07b	2.20±0.04a	2.06±0.06a
CN	2.36±0.07a	2.06±0.08a	1.96±0.04a
	——————ANOVA——————		
N levels (A)	*F* = 23.7690	*P* = 0.0000	
Years (B)	*F* = 17.9269	*P* = 0.0001	
A*B	*F* = 6.2815	*P* = 0.0024	
CV, %	8.00		

The total N application rates are 180 kg N ha^-1^ for RN and 240 kg N ha^-1^ for CN, respectively. RN: reduced nitrogen, CN: conventional nitrogen. Data are mean±S.D., different lower case letters in the same column means significant differences between RN and CN. Values under ANOVA are the F-test, probabilities (*P* value) and coefficient of variation of the sources of variation (LSD, *P* < 0.05).

### Root dry matter and distribution

Planting patterns affected crop root growth (S1 Table). The effect of planting pattern on maize root dry matter was not significant, but soybean root dry matter decreased significantly by 12.4% in IS compared with MS. Regarding N levels, both maize and soybean root dry matter increased in RN and CN compared with that in NN, whether in a monoculture or intercropping pattern; the difference between RN and CN was not significant.

**Fig 4:**
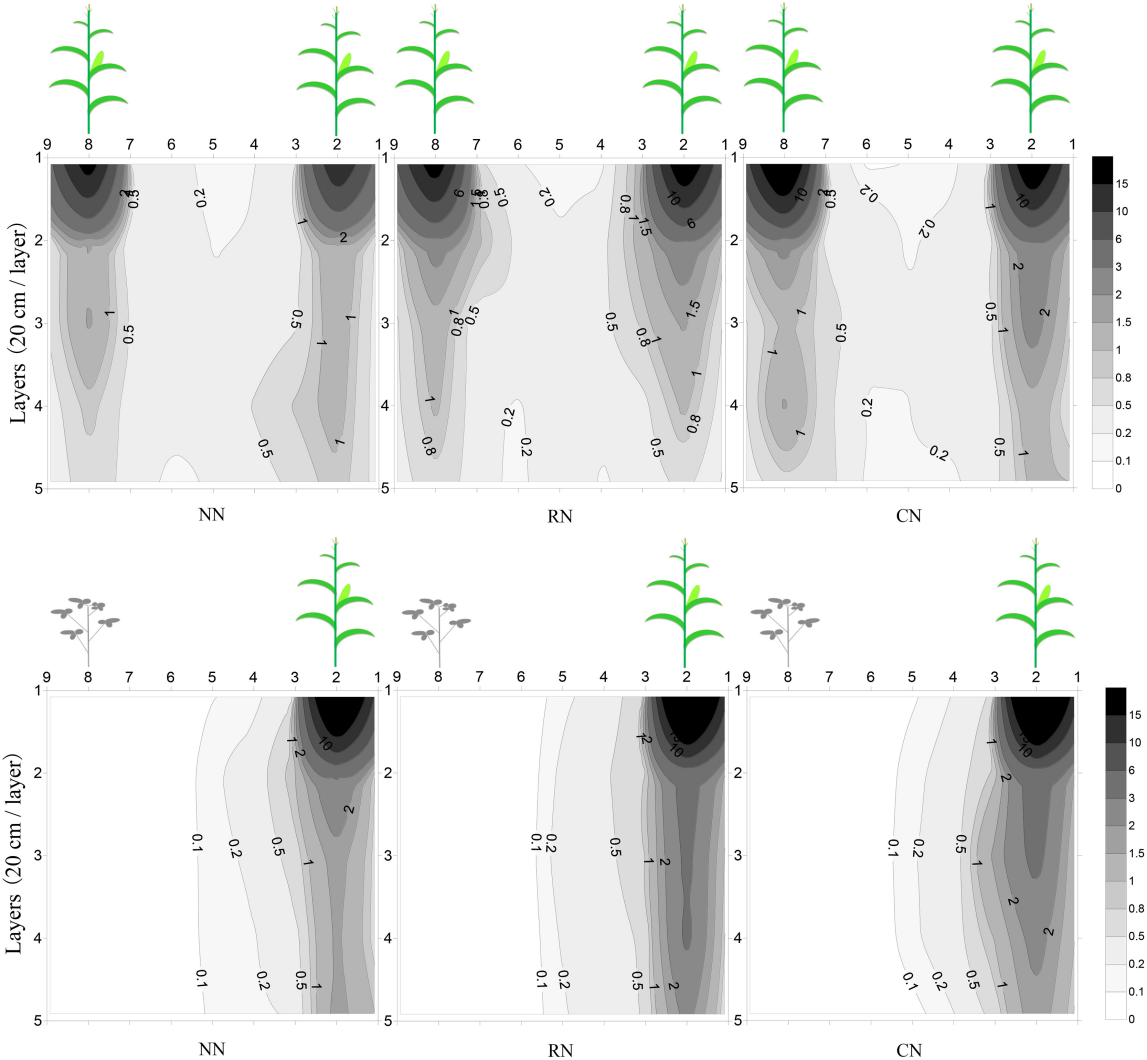
Effect of different below-ground interactions and N application rates on maize root distribution at the early grain-filling stage. MM with different N application rates (A), IM with different N application rates (B); the X-axis indicates depth (20 cm per layer) and the Y-axis indicates sampling interval (10 cm per interval); N application rates are 0, 180 kg N ha^-1^ (shared by soybean and maize), and 240 kg N ha^-1^ (180 kg N ha^-1^ for maize and 60 kg N ha^-1^ for soybean), respectively, the same as below.

**Fig 5:**
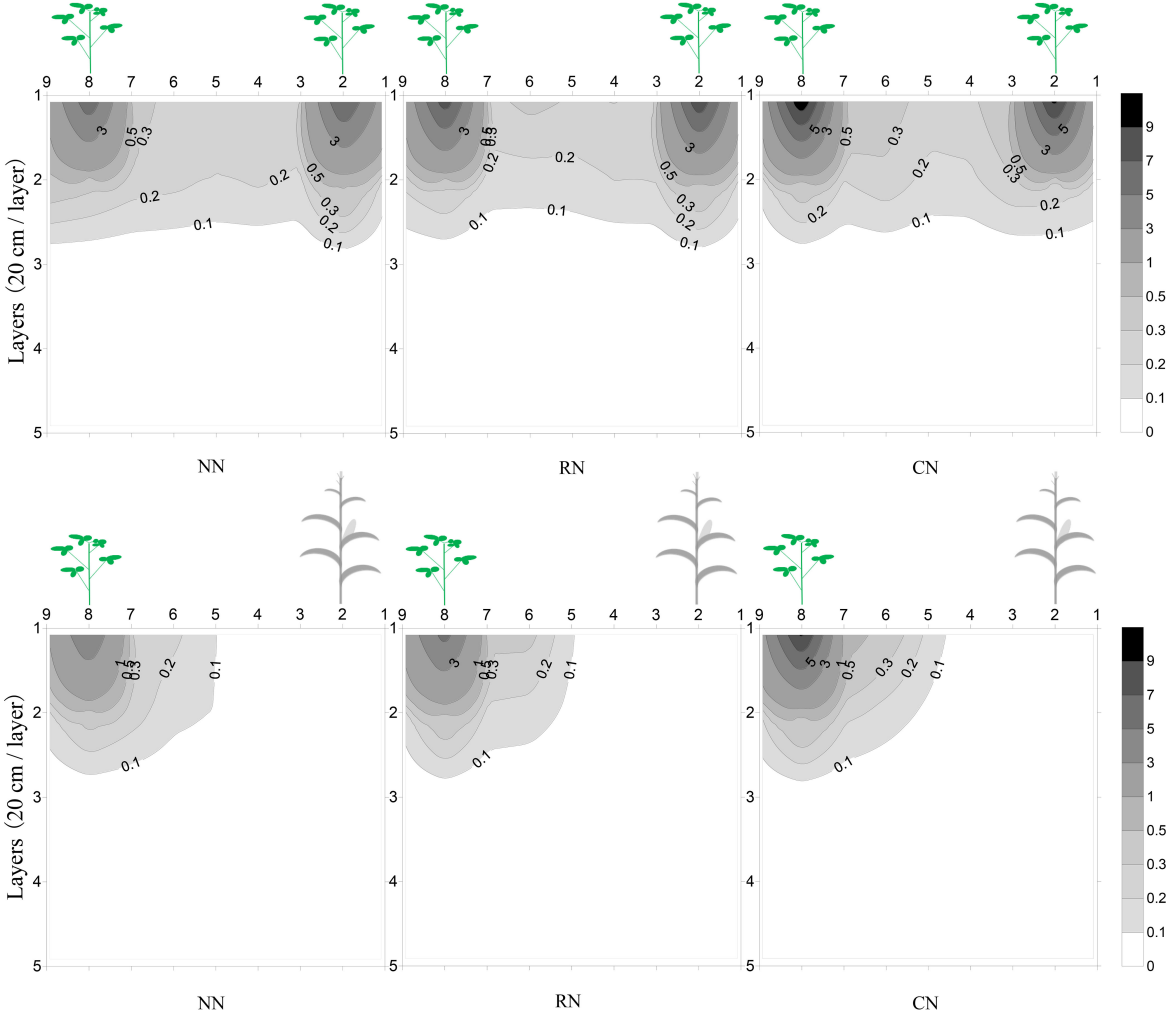
Effect of different below-ground interactions and N application rates on soybean root distribution at the R2 stage of development in 2014. MS with different N application rates (A), IS with different N application rates (B), the coordinate axis and N application rates were same as Fig 4.

Crop root growth was influenced by interspecific competition (Figs 4 and 5; S1 and S2 Tables). The first sampling was during the coexistence period when maize was at the early grain-filling stage and soybean was at the V3 stage of development. At that time, crop root dry matter increased with increased N input and crop root distribution displayed a similar trend. Compared with IM, maize root growth extended farther in the horizontal direction but fewer roots were distributed in the vertical direction under MM (Fig 4). With increased N application, the root dry matter of maize increased in both the upper 40 cm and at the 40–100 cm depth. The increase was particularly striking at the stem base in the upper 40 cm, and the maize root dry matter under IM rapidly increased at the stem base compared with that under MM. The maize root dry matter under IM also increased at the 20–80 cm depth from maize to soybean row (Fig 4).

During the coexistence period, soybean was at the V3 stage, and planting pattern and level of N significantly influenced the root growth of soybean (S2 Table). When soybean was at the V3 stage, the soybean root dry matter under IS was higher in CN than that in NN and RN (S2 Table). Under IS, soybean root dry matter declined compared with that in MS, but with increased N application, root dry matter increased and that of CN was higher than that of NN and RN (S2 Table). At the R2 stage of soybean, the root dry matter distribution under IS and MS was similar, with more roots at shallow depths, particularly at the stem base and in the upper 20 cm. The soybean root dry matter under IS increased with N application rates, but roots were less distributed both vertically and horizontally (Fig 5).

### Dry matter accumulation at the harvest stage

Planting patterns and N application rates had remarkable effects on crop grain yield (Table 2). From 2012 to 2014, straw dry matter and grain yield of IM declined by 3.4% and 3.5%, respectively, compared with those in MM; however, the difference was not significant. Compared with NN in IM, dry matter accumulation of RN and CN was significantly greater by 29.5% and 33.9% for straw dry matter and 19.6% and 26.6% for grain yield, respectively (Table 2). Effects of planting pattern and N level on soybean dry matter accumulation differed from those for maize. The straw dry matter and grain yield of MS increased and declined, respectively, compared with those in IS. Under different N application rates in MS, the grain yield of RN was greater than that in CN, whereas the straw dry matter of RN was lower. In IS, grain yield and straw dry matter of RN were greater than those of CN.

**Table 2:** Effect of planting patterns and N application rates on crop dry matter accumulation (Mg ha^-1^).

Years	N levels	Maize				Soybean				Relay intercropping	
		Straw		Grain		Straw		Grain		Total Grain	
		MM	IM	MM	IM	MS	IS	MS	IS	IM+IS	
2012	NN	6.52±0.18a	6.37±0.08a	6.17±0.20b	6.09±0.03c	2.26±0.08b	1.55±0.08b	1.55±0.05b	1.57±0.03c	7.66±0.04c	
	RN	6.76±0.26a	6.80±0.51a	6.91±0.20a	6.79±0.02a	2.77±0.15a	2.30±0.15a	1.89±0.07a	2.37±0.03a	9.15±0.02a	
	CN	6.61±0.20a	6.62±0.31a	7.19±0.15a	6.35±0.01b	2.20±0.02b	2.25±0.11a	1.47±0.04b	2.18±0.02b	8.52±0.04b	
2013	NN	6.35±0.12b	5.19±0.30b	6.29±0.42b	5.63±0.62b	2.32±0.08b	1.90±0.01b	1.68±0.05b	1.59±0.01b	7.22±0.61c	
	RN	6.74±0.20a	6.88±0.47a	7.90±0.40a	8.64±0.21a	2.99±0.10a	2.24±0.13a	1.98±0.21a	2.22±0.29a	10.76±0.15a	
	CN	6.43±0.15ab	6.24±0.51a	8.10±1.07a	7.84±0.18a	3.08±0.23a	2.00±0.05b	1.80±0.05ab	1.95±0.01a	9.78±0.18b	
2014	NN	3.31±0.14c	3.65±0.16b	5.60±0.35b	5.78±0.45b	2.90±0.12b	1.96±0.16b	2.35±0.10ab	2.18±0.13c	7.96±0.32c	
	RN	4.87±0.22b	5.45±0.66a	8.01±0.32a	8.01±0.24a	4.09±0.07a	2.63±0.04a	2.51±0.19a	2.66±0.08a	10.67±0.17a	
	CN	5.34±0.22a	4.90±0.26a	8.57±0.35a	7.46±0.15a	4.03±0.12a	2.47±0.09a	2.16±0.02b	2.36±0.02b	9.83±0.16b	
		——————————————————————————ANOVA——————————————————————————————————									
Cropping system (A)		*F* = 1.1320	*P* = 0.2944	*F* = 5.7591	*P* = 0.0217	*F* = 516.4056	*P* = 0.0000	*F* = 39.7325	*P* = 0.0000		
N level (B)		*F* = 50.9127	*P* = 0.0000	*F* = 119.4489	*P* = 0.0000	*F* = 133.7926	*P* = 0.0000	*F* = 78.7218	*P* = 0.0000	*F* = 41.3342	*P* = 0.0000
Year (C)		*F* = 213.1326	*P* = 0.0000	*F* = 21.9925	*P* = 0.0000	*F* = 174.9464	*P* = 0.0000	*F* = 134.7953	*P* = 0.0000	*F* = 240.9101	*P* = 0.0000
A*B		*F* = 4.1124	*P* = 0.0247	*F* = 6.4437	*P* = 0.0041	*F* = 3.0986	*P* = 0.0573	*F* = 20.2804	*P* = 0.0000		
A*C		*F* = 3.6301	*P* = 0.0366	*F* = 0.5561	*P* = 0.5783	*F* = 58.3170	*P* = 0.0000	*F* = 12.9331	*P* = 0.0001		
B*C		*F* = 10.8195	*P* = 0.0000	*F* = 11.1760	*P* = 0.0000	*F* = 7.7414	*P* = 0.0001	*F* = 3.3121	*P* = 0.0207	*F* = 13.9854	*P* = 0.0000
A*B*C		*F* = 3.5312	*P* = 0.0157	*F* = 2.4430	*P* = 0.0642	*F* = 14.1536	*P* = 0.0000	*F* = 1.8861	*P* = 0.1341		
CV, %		8.52		14.81		26.33		15.13		13.92	

The total N application rates are 0, 180 kg N ha^-1^ and 240 kg N ha^-1^, respectively. MM: monoculture maize, IM: intercropped maize, MS: monoculture soybean, IS: intercropped soybean; NN: no nitrogen, RN: reduced nitrogen, CN: conventional nitrogen. Data are mean±S.D., different lower case letters in the same column means significant differences. Values under ANOVA are the *F*-test, probabilities (*P* value) and coefficient of variation of the sources of variation (LSD, *P* < 0.05).

### Nitrogen uptake

Nitrogen application significantly increased N uptake under the different planting patterns (Table 3). The N uptake of straw and grain was 7.4% higher and 4.0% lower, respectively, in IM than in MM and 55.3% (significantly) higher and 8.5% lower, respectively, in MS than in IS. Comparing N application rates in MM, the N uptake of grain in CN was greater than that in RN, whereas the straw N uptake in RN was greater than that in CN. In IM, grain and straw N uptake in RN were higher than those in CN. The N uptake of soybean in RN was the highest among all treatments, with N uptake of straw and grain greater in RN than in CN for MS and IS.

**Table 3:** N uptake of maize and soybean under different N levels and planting patterns (kg ha^-1^).

Years	N levels	Maize				Soybean			
		Straw		Grain		Straw		Grain	
		MM	IM	MM	IM	MS	IS	MS	IS
2012	NN	49.4±1.7b	47.2±0.2b	65.1±2.8b	62.5±2.9c	20.7±0.6c	12.7±0.4c	96.5±3.6b	103.5±4.3c
	RN	55.4±1.8a	60.9±4.2a	77.9±3.2a	76.1±3.0a	28.4±0.3a	22.1±3.3a	130.2±3.7a	160.6±5.2a
	CN	50.6±3.0a	51.0±5.5b	79.9±1.2a	68.1±1.3b	24.1±1.7b	18.0±0.6b	100.3±3.7b	144.5±5.4b
2013	NN	44.0±0.4c	34.8±2.3b	75.2±3.5b	71.2±8.4b	29.7±1.1a	18.2±0.1b	134.6±4.0b	128.9±0.4c
	RN	56.8±0.9a	60.5±3.3a	110.4±8.0a	118.7±5.3a	32.4±3.3a	21.9±1.1a	163.6±8.7a	178.1±13.9a
	CN	51.7±4.2b	53.4±6.9a	119.5±15.9a	106.6±5.8a	32.2±1.3a	19.2±0.5b	142.5±5.1b	156.9±1.0b
2014	NN	16.2±1.9b	21.6±0.4b	46.7±7.1b	43.0±1.9c	34.2±2.9b	22.5±2.5b	154.8±5.3b	141.1±9.8c
	RN	33.4±0.6a	43.9±7.0a	89.3±4.7a	96.2±3.3a	51.2±2.0a	29.5±1.1a	173.5±9.6a	176.6±7.0a
	CN	36.5±2.6a	39.8±2.4a	95.3±11.5a	87.8±2.1b	50.2±3.1a	28.8±1.1a	144.6±1.6b	157.2±1.1b
		——————————————————————————ANOVA————————————————————————							
Cropping system (A)		*F* = 3.0829	*P* = 0.0876	*F* = 3.5191	*P* = 0.0688	*F* = 597.9729	*P* = 0.0000	*F* = 49.2350	*P* = 0.0000
N level (B)		*F* = 115.8064	*P* = 0.0000	*F* = 164.5326	*P* = 0.0000	*F* = 89.2205	*P* = 0.0000	*F* = 164.0866	*P* = 0.0000
Year (C)		*F* = 211.7739	*P* = 0.0000	*F* = 105.5390	*P* = 0.0000	*F* = 316.6694	*P* = 0.0000	*F* = 162.9293	*P* = 0.0000
A*B		*F* = 7.9545	*P* = 0.0014	*F* = 6.4252	*P* = 0.0041	*F* = 3.5793	*P* = 0.0382	*F* = 24.1328	*P* = 0.0000
A*C		*F* = 7.0742	*P* = 0.0026	*F* = 0.4479	*P* = 0.6425	*F* = 44.1959	*P* = 0.0000	*F* = 22.0533	*P* = 0.0000
B*C		*F* = 8.1159	*P* = 0.0001	*F* = 17.8079	*P* = 0.0000	*F* = 13.4760	*P* = 0.0000	*F* = 4.9729	*P* = 0.0027
A*B*C		*F* = 1.9747	*P* = 0.1193	*F* = 0.4916	*P* = 0.7419	*F* = 6.1005	*P* = 0.0007	*F* = 0.8894	*P* = 0.4802
CV, %		28.84		27.48		37.51		15.82	

The total N application rates are 0, 180 kg N ha^-1^ and 240 kg N ha^-1^, respectively. MM: monoculture maize, IM: intercropped maize, MS: monoculture soybean, IS: intercropped soybean; NN: no nitrogen, RN: reduced nitrogen, CN: conventional nitrogen. Data are mean±S.D., different lower case letters in the same column means significant differences. Values under ANOVA are the F-test, probabilities (*P* value) and coefficient of variation of the sources of variation (LSD, *P* < 0.05).

### Nitrogen utilization

With reduced N input, NUE significantly increased for all planting patterns, and the NUE of MM, IMS and MS was 32.1%, 103.7% and 545.8% greater, respectively, in RN than in CN (Table 4). Intercropping maize with soybean increased NUE; the NUE of IMS increased by 105.1% over that of MM, and the NUE of RN was 139.0% greater in IMS than in MM (Table 4).

**Table 4:** Effect of planting patterns and N application rates on nitrogen use efficiency (%) in the maize-soybean relay strip intercropping system.

N levels	2012			2013			2014		
	MM	MS	IMS	MM	MS	IMS	MM	MS	IMS
RN	14.0±1.6a	92.0±1.4a	52.1±4.0a	35.6±3.7a	70.4±10.4a	70.1±11.0a	44.3±2.3a	79.4±9.5a	65.6±3.7a
CN	8.9±0.9b	12.0±2.9b	23.3±4.6b	28.9±8.1a	17.4±6.9b	34.6±4.9b	38.3±4.4a	9.6±3.5b	35.5±4.1b
	———ANOVA———								
Cropping system (A)	*F* = 62.8788	*P* = 0.0000							
N level (B)	*F* = 506.5064	*P* = 0.0000							
Year (C)	*F* = 21.0712	*P* = 0.0000							
A*B	*F* = 132.4075	*P* = 0.0000							
A*C	*F* = 18.7850	*P* = 0.0000							
B*C	*F* = 1.3460	*P* = 0.2731							
A*B*C	*F* = 3.8721	*P* = 0.0102							
CV, %	63.10								

The total N application rates are 180 kg N ha^-1^ for RN and 240 kg N ha^-1^ for CN, respectively. MM: monoculture maize, IM: intercropped maize, MS: monoculture soybean, IS: intercropped soybean, IMS: maize-soybean relay intercropping; RN: reduced nitrogen, CN: conventional nitrogen. Data are mean±S.D., different lower case letters in the same column means significant differences between RN and CN. Values under ANOVA are the F-test, probabilities (*P* value) and coefficient of variation of the sources of variation (LSD, *P* < 0.05).

## Discussion

### Effects of belowground interactions on crop performance in maize-soybean relay intercropping

In this study, the increase in crop yield and land productivity with maize-soybean relay intercropping was confirmed. The LER of the maize-soybean relay intercropping varied from 1.85 to 2.36 during the cropping seasons (Table 1). Results were similar for summer soybean-spring maize relay intercropping with an increase in land output and an LER of the relay intercropping that varied from 1.38 to 1.59 [19]. In the U.S., the total grain yield is higher in winter wheat-spring soybean relay intercropping than that with monoculture [28], and in China, the LER of winter wheat-spring cotton relay intercropping ranged from 1.20 to 1.53 [29]. Interspecific competition for nutrient resources is well known to affect crop growth and grain yield in intercropping systems [7, 17, 25, 26]. Numerous studies show that belowground interactions and competition for nutrients play a key role in intercropping and relay intercropping [6, 13, 22, 30]. Lv et al. demonstrated that competition for nutrients is more important than aboveground competition for light in maize-soybean intercropping [30]. By contrast, Yang et al. found no difference in yield between treatments in which roots were separated or not in maize-soybean relay intercropping [31]. The different conclusions may be the result of the difference in coexistence periods, because the component crops in intercropping have a longer coexistence period than that in relay intercropping; therefore, the following crop in relay intercropping can benefit from the longer recovery period. In this study, the complementary ecological niches of crop roots were advantageous to aboveground growth in the maize-soybean relay intercropping system (Figs 4 and 5; Tables 1 and 2, S1 and S2 Tables). The separation of root ecological niches in intercropping systems avoids interspecific competition for nutrients; for example, the total yield of the maize/fava bean intercropping system was significantly higher than that of the wheat/fava bean intercropping system [2]. During the coexistence period, maize was at the grain-filling stage, whereas soybean seedlings were at the stage with 3 fully developed trifoliate leaves. Maize roots rapidly proliferated underneath the maize plants in the soil top layer (0–40 cm) in relay intercropping compared with that in monoculture. The roots of soybean seedlings were insufficiently developed and primarily distributed underneath the soybean plants. However, after maize harvest (at the full-bloom stage of soybean), the soil volume occupied by soybean roots was similar between relay intercropping and monoculture, likely caused by the changes in root distribution and morphologies in space and time in relay intercropping systems [32]. In previous studies, crop roots show plasticity in response to soil nutrients and the distribution of water. With maize growth, the roots of maize extended to the soybean strip and proliferated underneath soybean [22]. When roots of component crops intermingle with one another, the preceding crop suppresses the growth of roots and decreases the shoot biomass of the following crop [13]. However, with the regrowth of roots of the following crop after harvest of the preceding crop, and fertilizer use efficiency increases, ultimately increasing the grain yield of the following crop [6, 13]. Thus, an optimized root system is advantageous in the acquisition of soil nutrients and provides sufficient nutrition for plant shoot growth.

The belowground benefits contributed to recovery of aboveground growth, thereby improving soybean yield (Table 2). The recovery of growth in soybean is responsible for pod formation, which improves the grain yield of soybean in the relay intercropping system [15]. The N competition ratio (NCR) of soybean relative to maize was greater than 1, indicating a positive interspecific facilitation between component crops in the maize-soybean relay intercropping system (Fig 3). This result is consistent with that of a previous study in which the NCR of a legume was greater than that of maize, and therefore, interspecific complementation between the component crops in fava bean-maize intercropping increased total grain yield [2]. In the present study, the N uptake and NUE of IMS increased through interspecific facilitation. The loss of N by maize in IMS was compensated by a gain in soybean in IMS (Tables 2 and 3). The NUE of IMS increased continually and was higher than that of MS in 2013 and 2014. These results confirmed that after maize harvest, soybean roots recovered growth and proliferated in the soil layer that was previously occupied by maize. Additionally, planting patterns have a large influence on crop root growth and nutrients utilization. For example, the interspecific competition for nutrients is more important than competition for sunlight when the number of maize rows to soybean rows is 1:1 and the distance between soybean and maize is 30 cm [30]. The roots of maize extend to soybean rows, whereas the roots of soybean are distributed primarily underneath the soybean plants when the number of maize rows to soybean rows is 1:3 and the distance between soybean and maize is 30 cm [22]. With the number of maize rows to soybean rows 2:2 and the distance between soybean and maize 60 cm, interactions belowground become weaker than those in the forward intercropping systems [31]. However, no difference is observed in the total grain yield between maize-soybean relay intercropping and maize-soybean intercropping [30, 31].

The availability of nutrients in soil affects crop growth. Relay intercropping maize with soybean can decrease soil pH and increase acid phosphatase activity, thereby increasing maize P uptake and total grain yield [20]. Soybean is well known to increase soil N input, with a rate of N fixation that varies from 100 to 140 kg N ha^-1^ per year [33]. Relay intercropping that includes legumes can increase N input and maize yield [11]. However, Amuse et al. found that the improvement in N use and grain yield was primarily a result of the biological N fixation of legumes in the previous cropping season [11], which occurs because during the coexistence period, the growth of legume seedlings is insufficient to compete for N or facilitate with wheat in spring legume-winter wheat relay intercropping [11]. Soil mineral nutrients can be efficiently utilized in intercropping and relay intercropping systems. On one hand, crop root exudates can increase the availability of soil nutrients and use efficiency, e.g., legume exudates can increase soil P availability, whereas those of cereals can increase soil Fe and Zn availability [34]. On the other hand, cereal crops must acquire abundant inorganic N, resulting in decreases in soil N concentration, which can increase the biological nitrogen fixation ability of legume crops [35]. The decrease of soil N concentration is advantageous, because the “suppressing effects” of soil N on N fixation by legumes are alleviated [36]. Additionally, relay intercropping maize with soybean can promote N transfer from legume to nonlegume [18]. Notably, a recent study found that maize root exudates can promote soybean nodule formation and increase soybean biological nitrogen fixation in maize-soybean intercropping [37]. Therefore, a suitable component crop can increase grain yield by increasing soil nutrient availability and utilization efficiency in relay intercropping or intercropping. However, information on the role of rhizosphere processes in interspecific facilitation between component crops in relay intercropping systems remains limited and inconclusive. Long-term location tests are required to understand the development and mechanisms of interspecific facilitation in the maize-soybean relay intercropping system.

### Effect of reduced N on nitrogen use efficiency and total grain yield in maize-soybean relay intercropping

Although chemical nitrogen fertilizers have been used worldwide to increase grain yields in the most recent decades [38], excessive N input may lead to yield loss [20, 39]. Particularly in China, nitrogen fertilizer use has successfully achieved food security in recent decades [40]. However, this high input of N has resulted in serious environmental pollution, which is adverse to the development of sustainable agriculture [40], e.g., leading to water eutrophication, soil acidification and air pollution [38, 41–43]. Therefore, strategies for the efficient management of N are urgently required. Good et al. identified two ways to increase crop NUE, traditional breeding and transgenic technology [44]. However, cropping techniques are another equally important approach to achieve N efficiency, which can rapidly and further promote the grain yield of highly nutrient-efficient cultivars; for example, intercropping and relay intercropping are resource efficient and environment friendly cropping systems that can increase farm land productivity [20, 31, 45]. In the present study, the NUE was higher in RN than in CN in MM, and in MS, the NUE was significantly higher in RN than in CN. The grain yield of MM increased significantly in CN compared with that in RN, whereas the grain yield of soybean decreased significantly in CN compared with that in RN. The reason for the yield loss might be soil acidification, because multiple fertilizer inputs can result in declines in soil pH [41], in addition to declines in soil pH that can occur under intercropping soybean with maize [20]. In IMS, the NUE of RN was significantly higher than that of CN, and the maize grain yield was maintained in RN, and soybean grain yield and NUE increased (Tables 1 and 3). These results confirmed that reduced N input led to grain yield loss in MM, in contrast to reduced N supply leading to increased maize yield and NUE in IMS. The results in this study are consistent with those of Yang et al. and Wang et al. who found that relay intercropping can increase resource utilization and land productivity [10, 20] and are also consistent with observations that a reduced N supply can maintain crop yield [25], whereas excessive N leads to a decline in LER [46].

Aboveground growth is strongly influenced by belowground processes. Crop root systems have the key role in soil nutrient acquisition, with effects on nutrient balance, and improvements in root distribution increase the potential opportunities for nutrient use. Interspecific root interactions in different cropping systems are manifested as competition or facilitation [39]. A complementary root distribution is a prerequisite for high-yields in intercropping systems [2]. In the present study, the coexistence period between maize and soybean was approximately 8 weeks, with sampling at the grain-filling stage of maize and soybean at approximately the V3 stage, and the results showed that the ecological niche of crop roots was separated (Figs 4 and 5; S2 Table). In the coexistence period, with the increase in N levels, the roots of maize rapidly proliferated underneath the maize plants and in the middle soil layer (20–80 cm), whereas those of soybeans were primarily distributed underneath the soybean plants. Although the soybean seedlings were weak, the seedlings helped to improve soil nutrients and facilitate maize in relay intercropping [20], which is in contrast to a previous study in which legume seedlings were too weak to compete or facilitate with cereal in relay intercropping [11]. This contrast may be the result of different legumes species. The CR of soybean was stronger than that of maize, and soybean performed better under RN than under CN. In a similar study, the CR of alfalfa was stronger than that of maize, and the yield advantage was greater in maize-alfalfa intercropping than that in the corresponding monocultures [47]. Additionally, N fertilizer input was required in long-term maize-soybean relay intercropping (Tables 1 and 2, Fig 3). During the coexistence period, the demand for N was high in the maize grain-filling stage, and an insufficient supply of N would result in yield loss [41].

The NUE of IMS increased significantly in RN compared with that in CN (Table 4). Under RN treatment, the nitrogen demand of maize was met, which led to increased NUE and grain yield (Tables 1 and 3). This result is consistent with that of Hasegawa et al. [44]. Additionally, the N uptake of maize led to a decrease in soil N concentration. Gan et al. found that low N levels were advantageous, because the suppressing effect of N on biological nitrogen fixation was removed [42]. Similar results are reported that intercropping with legumes can increase N input and thereby reduce chemical N fertilizer supply [17, 30, 32]. Reduced inputs of N can significantly reduce the residual N in soils and decrease N emissions and leaching losses [43]. The advantage in intercropping is achieved by complementary use of inorganic and atmospheric N and reducing competition for inorganic N, as occurs in pea/barley intercropping [45]. Intercropping advantages also included alleviation of N acquisition and increased sharing between maize and pea when compared with unfertilized intercropping systems [40]. In the present study, the LER and SPI of IMS were higher in RN than in CN, which is consistent with Yang et al. who found that reduced N can increase LER and total grain yield [25]. However, the influence of rhizosphere processes on the effect of interspecific facilitation on biological nitrogen fixation in relay intercropping conditions remains inconclusive. Long-term field location tests are required to explore the mechanisms and changes that promote soybean biological nitrogen fixation in the maize-soybean relay intercropping system.

## Conclusions

Our results revealed a positive interspecific facilitation between maize and soybean in the maize-soybean relay strip intercropping system. The separation of the root ecological niche contributed to interspecific facilitation. The selection of the component crop for relay strip intercropping should consider the interspecific complementary characteristics in the case of grain yield loss. For a long-term maize-soybean relay strip intercropping system, N fertilizer input is required. The total grain yield of the maize-soybean relay intercropping system increased under RN compared with that under CN. Furthermore, the nitrogen use efficiency in RN increased notably compared with that in CN in long-term maize-soybean relay strip intercropping.

## Supporting information

S1 Fig Diagram of root distribution investigation in rhizo-boxes in 2014. Setting the maize stem base as origin, root distribution was investigated by hand digging from maize row to soybean row. Numbers above lines are distances from maize to soybean rows. The sample interval from maize base to soybean base in the grid is 10 cm long, while from crops to box edgd was 15 cm long, and soil blocks sampling width and depth were 38 and 20 cm.
(TIF)

S1 Table Root dry matter of crops under different N application rates in 2012 and 2013 (Mg ha^-1^). The total N application rates are 0, 180 kg N ha^-1^ and 240 kg N ha^-1^, respectively. MM: monoculture maize, IM: intercropped maize, MS: monoculture soybean, IS: intercropped soybean; NN: no nitrogen, RN: reduced nitrogen, CN: conventional nitrogen. Data are mean±S.D., different lower case letters in the same column means significant differences. Values under ANOVA are the F-test, probabilities (*P* value) and coefficient of variation of the sources of variation (LSD, *P* < 0.05).
(DOCX)

S2 Table Effect of different below-ground interactions and N application rates on soybean root dry matter accumulation (g/plant) at the V3 stage of development in 2014 (experiment 2). The total N application rates are 0, 180 kg N ha^-1^ and 240 kg N ha^-1^, respectively. MS: monoculture soybean, IS: intercropped soybean; NN: no nitrogen, RN: reduced nitrogen, CN: conventional nitrogen. Data are mean±S.D., different lower case letters in the same column means significant differences. Values under ANOVA are the F-test, probabilities (*P* value) and coefficient of variation of the sources of variation (LSD, *P* < 0.05).
(DOCX)
