# Supplementary figures and images for: Effects of reduced nitrogen inputs on crop yield and nitrogen use efficiency in a long-term maize-soybean relay strip intercropping system
Source: PLoS One. 2017 Sep 14;12(9):e0184503. doi: 10.1371/journal.pone.0184503 (PMC5598979; doi:10.1371/journal.pone.0184503)

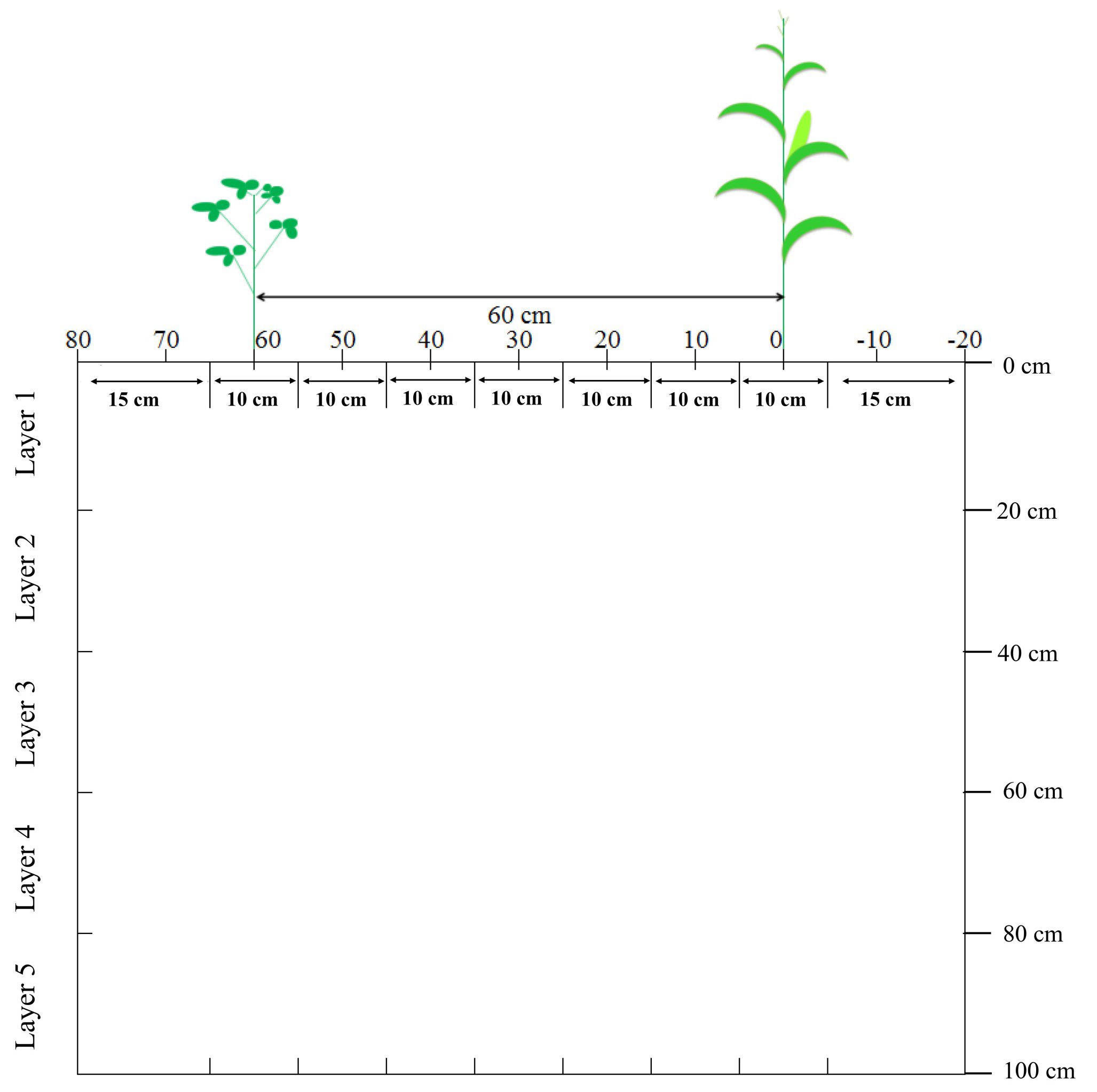

Supplement: S1 Fig — Setting the maize stem base as origin, root distribution was investigated by hand digging from maize row to soybean row. Numbers above lines are distances from maize to soybean rows. The sample interval from maize base to soybean base in the grid is 10 cm long, while from crops to box edgd was 15 cm long, and soil blocks sampling width and depth were 38 and 20 cm. (TIF) [file pone.0184503.s001.tif]
