# Supplementary material for: Effects of reduced nitrogen inputs on crop yield and nitrogen use efficiency in a long-term maize-soybean relay strip intercropping system
Source: PLoS One. 2017 Sep 14;12(9):e0184503. doi: 10.1371/journal.pone.0184503 (PMC5598979; doi:10.1371/journal.pone.0184503)
Supplement: S1 Table — The total N application rates are 0, 180 kg N ha-1 and 240 kg N ha-1, respectively. MM: monoculture maize, IM: intercropped maize, MS: monoculture soybean, IS: intercropped soybean; NN: no nitrogen, RN: reduced nitrogen, CN: conventional nitrogen. Data are mean±S.D., different lower case letters in the same column means significant differences. Values under ANOVA are the F-test, probabilities (P value) and coefficient of variation of the sources of variation (LSD, P < 0.05). (DOCX) [file pone.0184503.s002.docx]

**Supporting File**

**S1 Table**  **Root dry matter of crops under different N application rates in 2012 and 2013 (Mg ha^-1^).**

| N levels | 2012 | | | | 2013 | | | | | | | |
| --- | --- | --- | --- | --- | --- | --- | --- | --- | --- | --- | --- | --- |
|  | MM | IM | MS | IS | MM | | IM | | | MS | | IS |
| NN | 0.92±0.05b | 0.94±0.02b | 0.34±0.01b | 0.27±0.01b | 0.91±0.05b | | 0.94±0.09b | | | 0.37±0.02b | | 0.30±0.02b |
| RN | 1.16±0.07a | 1.13±0.03a | 0.42±0.00a | 0.39±0.01a | 1.17±0.06a | | 1.14±0.12a | | | 0.45±0.02a | | 0.39±0.03a |
| CN | 1.27±0.07a | 1.23±0.11a | 0.45±0.03a | 0.40±0.02a | 1.20±0.02a | | 1.26±0.08a | | | 0.46±0.03a | | 0.41±0.03a |
|  | -------------------------------ANOVA-------------------------------- | | | | |  | |  |  | |  | |
| Cropping system (A) | *F*=0.0002 | *P*=0.9890 | *F*=52.1153 | *P*=0.0000 |  | |  | | |  | |  |
| N level (B) | *F*=60.5816 | *P*=0.0000 | *F*=91.8093 | *P*=0.0000 |  | |  | | |  | |  |
| Year (C) | *F*=0.0868 | *P*=0.7708 | *F*=7.4254 | *P*=0.0118 |  | |  | | |  | |  |
| A*B | *F*=0.3198 | *P*=0.7294 | *F*=1.0538 | *P*=0.3642 |  | |  | | |  | |  |
| A*C | *F*=0.5130 | *P*=0.4808 | *F*=0.6590 | *P*=0.4249 |  | |  | | |  | |  |
| B*C | *F*=0.1217 | *P*=0.8860 | *F*=0.7453 | *P*=0.4853 |  | |  | | |  | |  |
| A*B*C | *F*=0.4662 | *P*=0.6330 | *F*=0.2590 | *P*=0.7739 |  | |  | | |  | |  |
| CV, % | 13.31 | | 15.37 | |  | |  | | |  | |  |

The total N application rates are 0, 180 kg N ha^-1^ and 240 kg N ha^-1^, respectively. MM: monoculture maize, IM: intercropped maize, MS: monoculture soybean, IS: intercropped soybean; NN: no nitrogen, RN: reduced nitrogen, CN: conventional nitrogen. Data are mean±S.D., different lower case letters in the same column means significant differences. Values under ANOVA are the F-test, probabilities (*P* value) and coefficient of variation of the sources of variation (LSD, *P* < 0.05).
