# Supplementary material for: Effects of reduced nitrogen inputs on crop yield and nitrogen use efficiency in a long-term maize-soybean relay strip intercropping system
Source: PLoS One. 2017 Sep 14;12(9):e0184503. doi: 10.1371/journal.pone.0184503 (PMC5598979; doi:10.1371/journal.pone.0184503)
Supplement: S2 Table — The total N application rates are 0, 180 kg N ha-1 and 240 kg N ha-1, respectively. MS: monoculture soybean, IS: intercropped soybean; NN: no nitrogen, RN: reduced nitrogen, CN: conventional nitrogen. Data are mean±S.D., different lower case letters in the same column means significant differences. Values under ANOVA are the F-test, probabilities (P value) and coefficient of variation of the sources of variation (LSD, P < 0.05). (DOCX) [file pone.0184503.s003.docx]

**Supporting File**

**S2 Table**  **Effect of different below-ground interactions and N application rates on soybean root dry matter accumulation (g/plant) at the V3 stage of development in 2014 (experiment 2).**

| N levels | Dry weight (g/plant ) | |
| --- | --- | --- |
|  | MS | IS |
| NN | 0.14±0.01b | 0.11±0.01b |
| RN | 0.16±0.01ab | 0.13±0.00b |
| CN | 0.18±0.01a | 0.16±0.01a |
|  | -----------------ANOVA---------------- | |
| Cropping system (A) | *F*=18.4437 | *P*=0.0051 |
| N levels (B) | *F*=20.3397 | *P*=0.0021 |
| A*B | *F*=0.9932 | *P*=0.4240 |
| CV, % | 15.32 | |

The total N application rates are 0, 180 kg N ha^-1^ and 240 kg N ha^-1^, respectively. MS: monoculture soybean, IS: intercropped soybean; NN: no nitrogen, RN: reduced nitrogen, CN: conventional nitrogen. Data are mean±S.D., different lower case letters in the same column means significant differences. Values under ANOVA are the F-test, probabilities (*P* value) and coefficient of variation of the sources of variation (LSD, *P* < 0.05).
